# The respiratory and fecal microbiota of beef calves from birth to weaning

**DOI:** 10.1128/msystems.00238-24

**Published:** 2024-06-20

**Authors:** Muhammed Salah Uddin, Jose Ortiz Guluarte, Matthew Waldner, Trevor W. Alexander

**Affiliations:** 1 Lethbridge Research and Development Centre, Agriculture and Agri-Food Canada, Lethbridge, Alberta, Canada; 2 Department of Agricultural, Food and Nutritional Science, University of Alberta, Edmonton, Alberta, Canada; University of California San Diego, La Jolla, California, USA

**Keywords:** beef calf, birth to weaning, bovine respiratory disease, respiratory microbiota, fecal microbiota

## Abstract

The development and growth of animals coincide with the establishment and maturation of their microbiotas. To evaluate the respiratory and fecal microbiotas of beef calves from birth to weaning, a total of 30 pregnant cows, and their calves at birth, were enrolled in this study. Deep nasal swabs and feces were collected from calves longitudinally, starting on the day of birth and ending on the day of weaning. Nasopharyngeal, vaginal, and fecal samples were also collected from cows, and the microbiotas of all samples were analyzed. The fecal microbiota of calves was enriched with *Lactobacillus* during the first 8 weeks of life, before being displaced by genera associated with fiber digestion, and then increasing in diversity across time. In contrast, the diversity of calf respiratory microbiota generally decreased with age. At birth, the calf and cow nasal microbiotas were highly similar, indicating colonization from dam contact. This was supported by microbial source-tracking analysis. The structure of the calf nasal microbiota remained similar to that of the cows, until weaning, when it diverged. The changes were driven by a decrease in *Lactobacillus* and an increase in genera typically associated with bovine respiratory disease, including *Mannheimia*, *Pasteurella*, and *Mycoplasma*. These three genera colonized calves early in life, though *Mannheimia* was initially transferred from the cow reproductive tract. Path analysis was used to model the interrelationships of calf respiratory and fecal microbiotas. It was observed that respiratory *Lactobacillus* and fecal *Oscillospiraceae* UCG-005 negatively affected the abundance of *Mannheimia* or *Pasteurella*.

**IMPORTANCE:**

In beef cattle production, bovine respiratory disease (BRD) accounts for most of the feedlot morbidities and mortalities. Metaphylaxis is a common management tool to mitigate BRD, however its use has led to increased antimicrobial resistance. Novel methods to mitigate BRD are needed, including microbiota-based strategies. However, information on the respiratory bacteria of beef calves prior to weaning was limited. In this study, it was shown that the microbiota of cows influenced the initial composition of both respiratory and fecal microbiotas in calves. While colonization of the respiratory tract of calves by BRD-associated genera occurred early in life, their relative abundances increased at weaning, and were negatively correlated with respiratory and gut bacteria. Thus, microbiotas of both the respiratory and gastrointestinal tracts have important roles in antagonism of respiratory pathogens and are potential targets for enhancing calf respiratory health. Modulation may be most beneficial, if done prior to weaning, before opportunistic pathogens establish colonization.

## INTRODUCTION

Bovine respiratory disease (BRD) affects the health of beef calves, and accounts for losses due to treatment, prevention, and reduced animal productivity costs (1). Cattle are most susceptible to BRD during feedlot placement, with the majority of cases occurring within the first 60 days of arrival (2); however, young calves on farm can also be affected. Primary viral infections or stressors, that include weaning and shipping, are proposed to reduce host immunity during the transition to feedlots (3). Consequently, opportunistic bacterial pathogens residing in the upper respiratory tract can translocate to the lungs causing bronchopneumonia (4). As a result, cattle determined to be at risk for BRD are frequently administered antimicrobials upon feedlot entry (5). However, recent reports indicate high levels of antimicrobial resistance in BRD pathogens from feedlot cattle, potentially limiting the therapeutic efficacy of antimicrobials (6, 7). Thus, novel methods to reduce feedlot antimicrobial use and the development and spread of resistance are needed.

The composition of the bovine airway microbiota evolves over time due to a variety of selection pressures that influence colonization of the respiratory tract. These include endogenous forces such as mucus, IgA, innate/adaptive immune recognition, and gut communication (8), and exogenous forces such as diet (9), infection (10), transportation stress (11), and parenteral antibiotics (12). There is increasing awareness regarding the importance of the mammalian microbiome in relation to health, and it has become clear that the resident microbiota of the respiratory tract have a critical role in preventing colonization by pathogens (13). Establishment and stability of the respiratory microbiota are important for homeostasis while disruption can lead to pathogenesis (14). Additionally, studies have shown that the respiratory microbiota and resilience to infection are influenced by signals from the gut (8). This occurs through the gut-lung axis in which gut metabolic products and immune cells migrate to the lungs. In calves, it was shown that feed supplemented with a postbiotic modulated innate immune function in pre-weaned dairy calves and ameliorated BRSV infection (15), highlighting a potential role between gut microbiota and respiratory immunity. Thus, critical to cattle health, is the prevalence of commensal and pathogenic microbiota in the respiratory and gastrointestinal tract, and factors that affect their abundance.

The respiratory microbiome consists of commensals and pathogens that affect host health. The main bacterial pathogens implicated in BRD are *Mannheimia haemolytica*, *Pasteurella multocida*, *Histophilus somni*, and *Mycoplasma bovis*. In recent studies on the bovine microbiome, it was shown that lactic acid-producing bacteria were possibly involved in colonization resistance against BRD pathogens (10, 16). In support of this, *Lactobacillus* probiotics isolated from the respiratory tract of feedlot cattle were capable of inhibiting the BRD pathogen *M. haemolytica in vivo* after intranasal inoculation (17). More recently, these same probiotics were shown to modulate the respiratory microbiota of auction market calves up to 42 days after intranasal administration (18). However, the probiotic strains were transient and did not establish colonization in the upper respiratory tract, likely due to being outcompeted by resident microbiota. Thus modulation of the bovine respiratory microbiota may be possible, but successful colonization and optimizing efficacy of probiotics may depend on when they are administered. Indeed, successful colonization by external bacteria may be enhanced before the microbiota of calves is established. In this regard, there is a lack of information on how the respiratory microbiota evolves after birth in beef calves, and when the microbiota matures. The purpose of this study was to characterize changes in the respiratory microbiota of beef calves from birth to weaning, and to investigate the sources of microbiota colonizing calves. The fecal microbiota was also investigated, given the potential importance of gastrointestinal bacteria and host respiratory health.

## MATERIALS AND METHODS

The study was approved by the Canadian Food Inspection Agency Animal Care Committee, and the welfare of the animals was maintained as per the standards established by the Canadian Council of Animal Care (19).

### Study design and sampling

Thirty synchronized pregnant cows (Angus × Herford, *Bos taurus*) from the Animal Diseases Research Institute (ADRI) herd in Lethbridge, Alberta, and their 30 calves at birth were enrolled in the study. Synchronization was used to shorten the calving interval and reduce variation in calf age. The cows were equally weighted by parity (*N* = 10 for parities 1, 2, or >2). The ADRI herd is a closed herd that was established in 1984. It is free from various pathogens, including bovine viral diarrhea virus, bovine herpes virus-1, *Leptospira* (serovars Canicola, Pomona, Hardjo, Grippotyphosa, and Copenhageni), *Anaplasma phagocytophilum*, bluetongue virus, and *Brucella abortus*. The herd undergoes annual testing for these pathogens, biannual testing for *Mycobacterium avium* subspecies paratuberculosis and bovine leukosis virus, and testing every 5 years for *Mycobacterium bovis*. Cattle that test positive for any of these disease agents are removed from the herd. None of the cattle used in the study were given antimicrobials or vaccines before or during the study. From days 1 to 56, cow/calf pairs were housed in outdoor pens exposed to ambient conditions, according to parity (*N* = 3 pens). This was done to facilitate the sampling of calves at a young age. During this time period, animals had *ad libitum* access to water and feed (silage-hay mixture). After day 56, cow-calf pairs were transferred to a single enclosed field and remained there for the rest of the study.

In this study, the calving day was considered study day 1, both for cows and calves. Nasopharyngeal (NP) samples were collected from cows on days 1, 56, and 180. Prior to obtaining a sample, the nostril was wiped clean thoroughly with a 70% ethanol solution. Extended guarded swabs (measuring 27 cm) with a rayon bud (MW 124, Medical Wire & Equipment, Corsham, England) were used for NP sampling (11). Vaginal samples were collected on the day of calving (day 1) and on day 56 using the same double-guarded swab. Swab tips were then cut and placed into sterile 1.5 mL tubes kept on ice. Subsequent to the vaginal sample collection, fecal samples of the cows were obtained on days 1 and 56, by digital palpation. Calf deep nasal swab samples were collected on days 1, 3, 7, 21, and 56, using sterile regular-size nylon flocked swabs (ESwab 480C, Copan, Murrieta, CA, USA) and on day 180 using the same swab used for cows. Fecal samples were collected from calves on days 3, 7, 21, 56, and 180. All calves were born within a span of 10 days, and each sampling date is precisely aligned with their actual date of birth, except for the day they were weaned (day 180). On this particular day, all calves underwent sampling before being weaned, which took place on a single day when they were approximately 180 days old. All the samples were transported to the lab and stored at −80°C within 1 h of collection.

### DNA extractions and amplicon sequencing

The extraction of nucleic acids from NP swabs was carried out using methods described earlier (11). DNA extraction from the vaginal swabs followed the same protocol. To obtain DNA from fecal samples, the feces were first freeze-dried and then mixed with a ball grinder. A total of 250 mg of the homogenized fecal material was used for the DNA extraction process and followed the manufacturer’s guidelines (Cat# 47016, DNeasy PowerSoil Pro Kit, QIAGEN). From all samples, DNA was used to amplify the V4 region of the 16S rRNA gene using primers 515-F (5′-GTGYCAGCMGCCGCGGTAA-′3) and 806-R (5′-GGACTACNVGGGTWTCTAAT-′3) (12). The MiSeq Reagent Kit v2 was used to sequence the amplicon on a MiSeq instrument (PE250) with 25,000 read coverage at Genome Quebec in Montreal, Quebec, Canada.

### Sequence processing, bioinformatics, and statistical analysis

Data quality assessment on the unprocessed reads was executed using FastQC v0.11.9 and MultiQC v1.12 (20). Sequence trimming was conducted using Trimmomatic v0.39 (21). Trimmomatic eliminated primer sequences and low-quality segments of reads, employing the following parameters: HEADCROP:10, SLIDINGWINDOW:5:20, LEADING:20, and TRAILING:20. Subsequent statistical analyses were carried out using R version 4.2.2 (22). Following sequence trimming, the paired reads underwent additional filtering utilizing the DADA2 v1.22.0 “filterAndTrim” function with default parameters and subsequently merged. Bimeric sequences were excised from the merged data set using the DADA2 “removeBimeraDenovo” function. Taxonomic classification of the 16S sequences was achieved utilizing DADA2’s “IdTaxa” function and the SILVA 138 database (23), which created the amplicon sequence variants (ASVs) table. Data manipulation was conducted using Phyloseq v1.42.0 (24), and figures were generated with ggplot2 v3.4.1 (25). The α-diversities were computed via “vegan” v2.6-4 (26) in the form of the Shannon diversity index and richness, and corresponding plots were generated. To reduce noise in downstream analysis, the ASV table was filtered to retain ASVs present with a count of at least two in a minimum of 1% of the samples. One-way analysis of variance (ANOVA) was employed to assess α-diversity with respect to treatment and time. The package “vegan” was used to calculate β-dispersion and permutational multivariate analysis of variance (PERMANOVA) with 9,999 permutations to examine the effects of treatment at each sampling time on the structure of the microbial community. β-diversity was calculated from the filtered ASV counts after they were subjected to size factor normalization using “GMPR” within DESeq2 v1.40.2 (27). Sample-to-sample distances were determined with the Bray-Curtis metric through Phyloseq ordination and plotted as a detrended correspondence analysis (DCA). The most abundant phyla and genera within each treatment and sample time were additionally calculated and plotted. Microbiota composition was analyzed with analysis of similarities (ANOSIM) using functions adapted from vegan 2.6-4. ANOSIM was performed on weighted and unweighted Unifrac distance matrices calculated from select sample groups.

Differential abundance analysis of taxonomic counts was performed with glmmSeq 0.5.5 (28). The analysis fit a general linear mixed model (GLMM) to an equation of “~day sampled + (1 | animal ID),” to investigate changes in the calf nasal or calf fecal microbiome over time. The GLMM was fit to the equation “~sample_group + day_sampled + (1 | animal_ID) + (1 | cow_calf_pair_ID) + sample_group * day_sampled” for analyses of paired treatments. The identifier of each animal (animal_ID) and the shared identifier between a cow-calf pair (cow_calf_pair_ID) acted as the random effect terms in the equation. Counts of the lowest identifiable taxonomic level for all ASVs were used as input to the GLMM to allow for the most precise model possible; however, only genus-level taxa are presented. Counts were transformed by taking the square root of the raw counts as this was determined to produce the most accurate models through testing of the following count transformations: raw; logarithm base *n*, 2, 10; the square root; and the cubed root. *Q*-values presented are adjustments of the *P* value that incorporate multiple testing corrections to control for the false discovery rate, with a *Q*-value of less than 0.05 considered significant.

An analysis was performed to model the relationships between three BRD-associated genera (*Mycoplasma*, *Mannheimia*, and *Pasteurella*), and 17 other genera in the calf microbiome during development. These genera were selected by taking the union of the 10 most relatively abundant genera from the calf nasal and calf fecal samples. A Pearson variance-covariance matrix was constructed of the relative abundances of these genera per sample, as well as two other variables (day, representing numeric sampling day; and sample type, a binary representation of calf nasal [1] or calf fecal [0] sample groups). The lavaan 0.6.14 package (29) was used to conduct the analysis with regression upon the variance-covariance matrix with dependent variables of day, sample type, *Mannheimia*, *Mycoplasma*, and *Pasteurella*. These three genera were selected as dependent variables considering their importance in BRD. The remaining 17 genera represented the independent variables for each regression. Variance of the dependent variables and covariance between the dependent variables was additionally calculated as part of the analysis. Significant (*P* < 0.05) relationships were plotted with tidySEM 0.2.3 (30).

### Source tracking analysis of the calf microbiota

The percentage of microbiota attributable to maternal (cow fecal, nasal, and vaginal) or alternate internal sources (calf fecal or nasal) within the calf nasal and calf fecal (sink) samples were calculated with a fast expectation-maximization microbial source tracking (FEAST) package (v1.10.0) in R as described previously (31). FEAST analysis was performed on count tables rarified to 3,000 reads per sample with default settings. Data were analyzed on a by-day basis. Calf nasal and fecal data used as sinks and sources were analyzed for all five presented days (3, 7, 21, 56, and 180). Cow vaginal source data exclusively came from day 1. In cases where cow nasal (days 1, 56, and 180) and cow fecal (days 1 and 56) source data were not available on the same day, the analysis used the data of the most recently sampled previous day as the source.

## RESULTS

### 16S rRNA gene sequencing overview

The unfiltered ASV table contained 45,453 unique ASVs from 16,886,370 merged paired reads in the 548 samples. The median sequences per sample were 30,345 ± 11,487.5, while the minimum sequences per sample were 6,113 and the maximum were 85,318. After filtering, the ASV table contained 3,102 ASVs from 12,642,451 merged paired reads in the 548 samples. The medium number of reads reduced to 23,425 ± 9,004.8 per sample, with a minimum of 4,296 and a maximum of 50,736.

### The community structure of cow and calf microbiota

**Fig 1:**
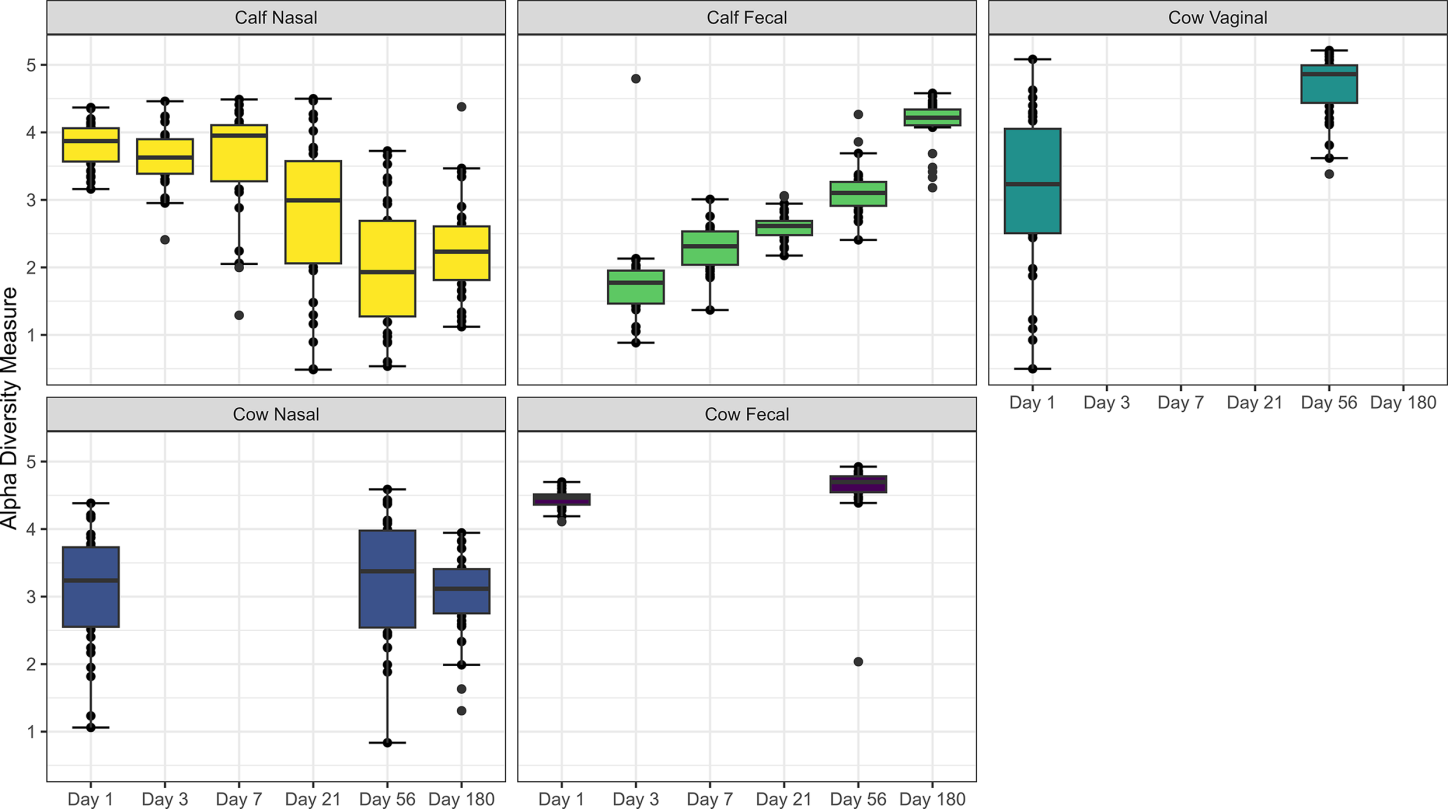
Alpha diversity of microbiota in calf and cow samples. The Shannon index was determined in calf (*N* = 30) deep nasal swabs and feces, as well as in cow (*N* = 30) nasopharyngeal, fecal, and vaginal samples over 180 days. Day 1 was the day of birth. Error bars indicate ±standard error of the mean. The box in the plots indicates the interquartile range (IQR) (middle 50% of the data), the middle line represents the median value, and the whiskers represent 1.5 times the IQR.

The Shannon diversity index was not affected by parity (*P* > 0.05), but was affected by time, sample type, and an interaction between time and sample type (*P* < 0.001, Fig. 1). In calf nasal samples, microbial diversity decreased by day 56 and remained lower at day 180, compared to day 1 samples (*P* < 0.05). In contrast, diversity increased over time in calf fecal samples, and was higher on Ddays 21, 56, and 180, compared to day 3 samples (*P* < 0.05). The increase in diversity of calf fecal samples led to their Shannon indices becoming more similar to those of cow fecal samples. For all cow sample types, Shannon diversity remained relatively constant across time.

**Fig 2:**
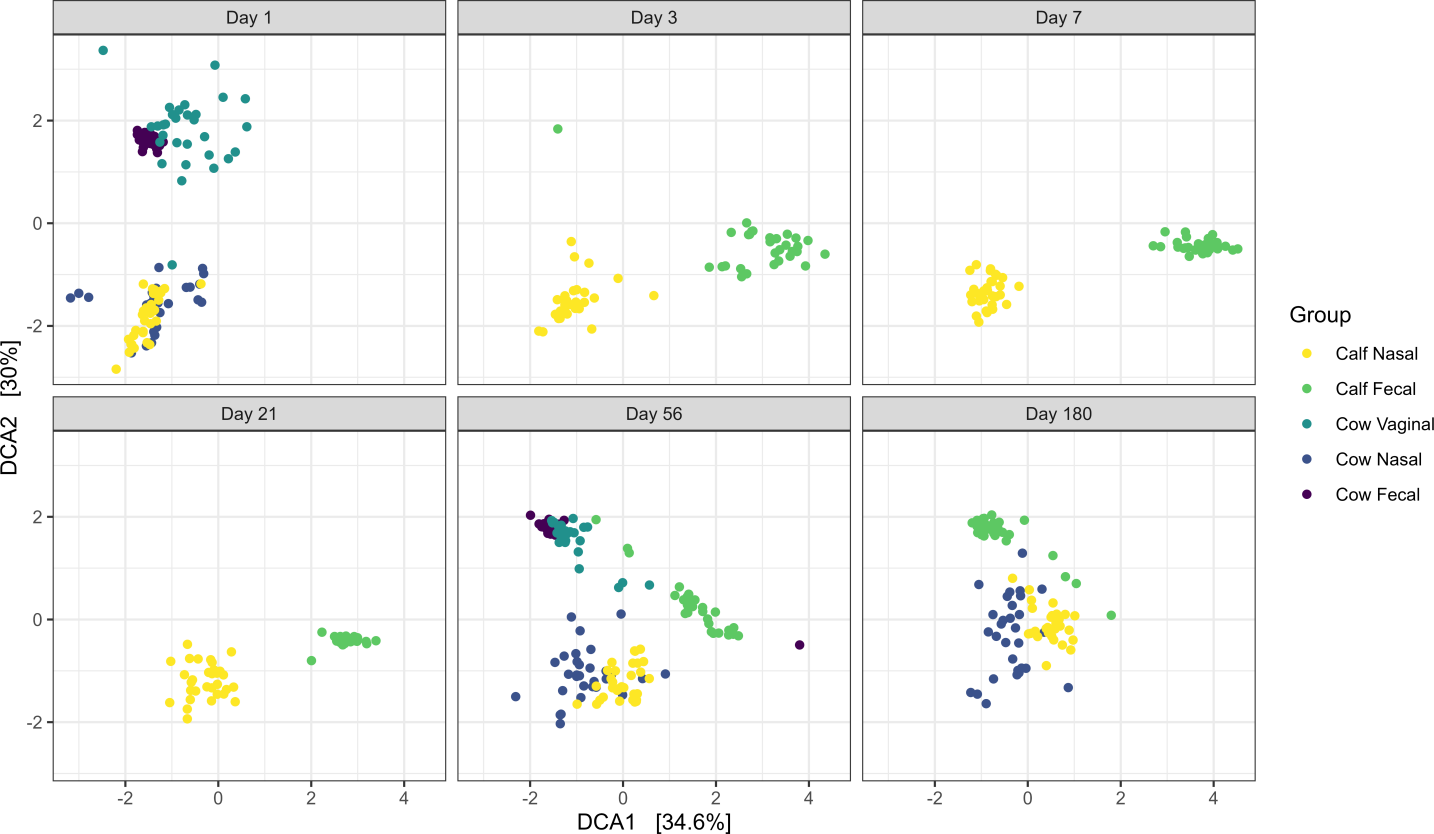
Detrended correspondence analysis (DCA) of the Bray-Curtis metric for microbiota in calf and cow samples. Calf (*N* = 30) deep nasal swabs and feces, as well as cow (*N* = 30) nasopharyngeal, fecal, and vaginal samples were analyzed over 180 days. Day 1 was the day of birth. The percentages of variation explained by the DCA are indicated on the axes.

β-Dispersion analysis indicated differences between the dispersion of groups, sampling times, and a combination of both (*P* < 0.001). A Turkey HSD test was performed for the combination of sampling day and group as confirmation that comparisons of group/day combinations had significant differences in dispersion. PERMANOVA revealed that the β-diversity representing the microbiota structure was not affected by cow parity (*P* = 0.31, *R*^2^ = 0.003) but was affected by the sampling day (*P* < 0.0001, *R*^2^ = 0.06246), the treatment group (*P* < 0.0001, *R*^2^ = 0.10452), and the interaction of sampling day and group (*P* < 0.0001, *R*^2^ = 0.07265). This was supported by clustering observed in DCA plots, which highlighted grouping by treatment group (Fig. 2). The microbial structure of calf feces shifted over time and clustering of these samples became closer in proximity to cow fecal samples. In contrast, clustering of calf nasal samples became less clustered to cow nasal samples on day 180. For vaginal samples, clustering was closest to cow fecal microbiota. Because alpha and beta diversity metrics were not affected by parity, parity was not evaluated in any other analysis.

### Microbiota source tracking and ANOSIM

**Fig 3:**
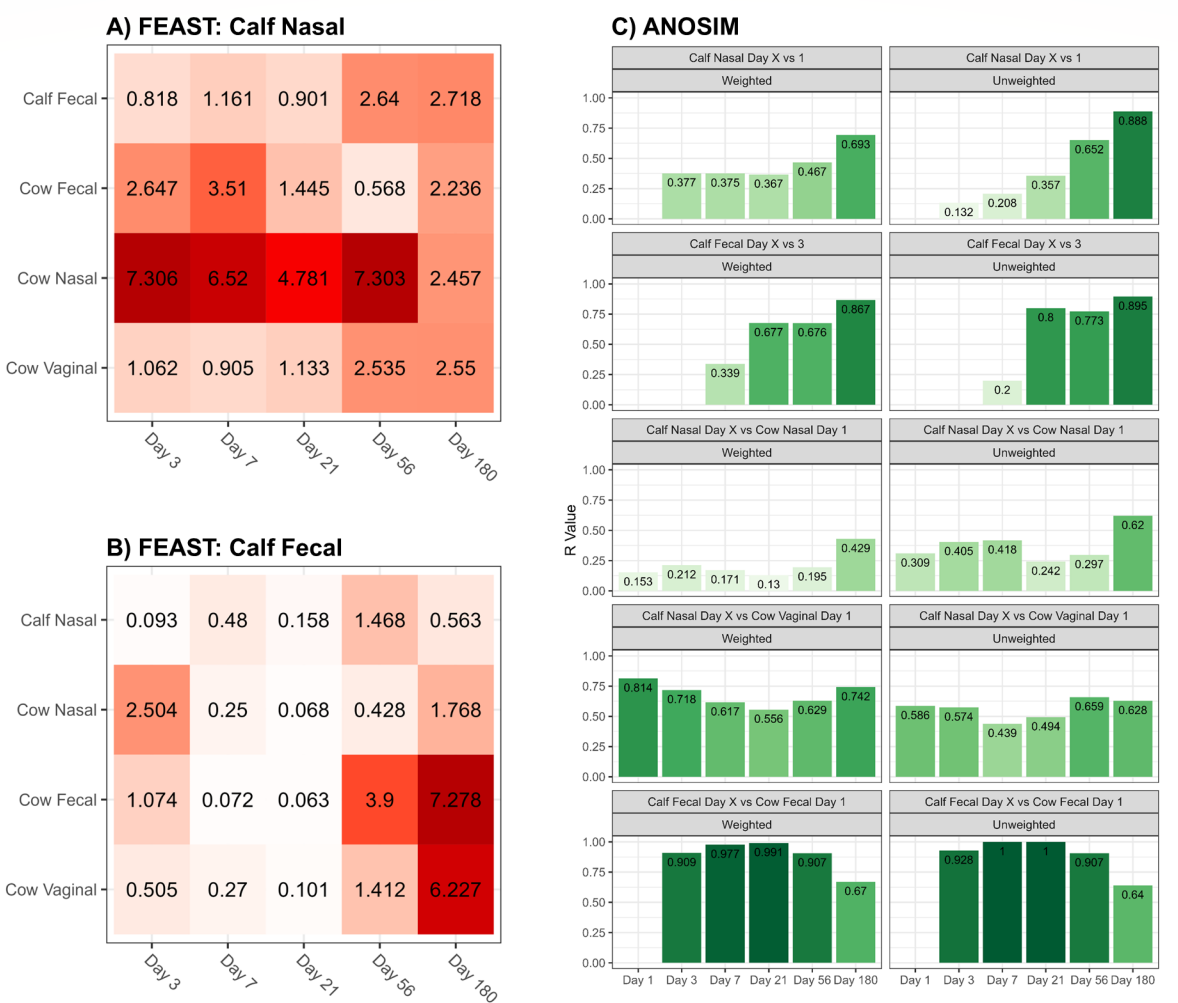
Microbial source tracking and analysis of similarities. (**A**) Percentage of calf nasal microbiota attributable to other sources estimated by fast expectation-maximization microbial source tracking (FEAST) analysis. FEAST was performed by sampling day and used each of the calf data sets as the sink variable. (**B**) Percentage of calf fecal microbiota attributable to other sources estimated by FEAST analysis. (**C**) Analysis of similarities (ANOSIM) results for the comparison of microbiota compositions in different sample types and time points using both weighted and unweighted UniFrac distance beta diversity. The *R*-value served as the test statistic, ranging from 1 to 0. An *R*-value close to 1 indicates a significant difference between groups, while an *R* close to 0 suggests that the groups are similar.

To track the sources of calf nasal and fecal microbiota, we used the fast expectation-maximization microbial source tracking (FEAST) algorithm. For the calf nasal microbiota source tracking, the largest contribution was attributed to cow nasal microbiota with a range of 2.457–7.306% across sampling time points, followed by cow fecal microbiota ranging from 0.568% to 3.51% across sampling time points (Fig. 3A). On day 3, cow nasal microbiota was the highest contributor (7.306%) to calf nasal microbiota and it remained the same for most of the sampling time points. Among the cow niches evaluated, the cow vaginal microbiota contributed the least to calf nasal bacteria. Three days after birth, microbiota of the cow’s nasopharynx (2.504%) was the greatest contributing source of bacteria in calf feces (Fig. 3B). Over time, cow fecal microbiota (7.278% on day 180) was the greatest contributor of microbiota in calf feces.

The calf nasal and fecal microbiota became increasingly dissimilar across time when compared to the respective first samples collected, for both weighted and unweighted ANOSIM comparisons (Fig. 3C). The differences in calf versus cow respiratory bacteria for the first 56 days accounted for less than 21% of the variation between the microbiotas (weighted *R* = 0.13–0.212; *P* < 0.01). However, on day 180, the calf and cow dissimilarity increased (unweighted *R* = 0.429; *P* < 0.001). In contrast, when fecal microbiota were compared between calf and cow groups, the microbiotas were most dissimilar the first 56 days (weighted *R* = 0.907–0.991; *P* < 0.01) before increasing in similarity on day 180 (unweighted *R* = 0.67; *P* < 0.001). Over time, the similarity of calf nasal microbiota compared to cow vaginal microbiota remained relatively constant, and was lower than that for cow versus calf nasal microbiotas.

### Composition of the microbiota

Across sample type and time, a total of 22 phyla were identified. The five most common phyla were similar for each sample type, but varied in relative abundance (Table S1). For calf and cow nasal samples, Proteobacteria and Actinobacteria were most abundant, while Firmicutes dominated the microbiota of calf and cow fecal samples, as well as cow vaginal samples. In total, 669 genera were observed across all samples and time points, with 285, 128, 584, 180, and 405 being detected in calf nasal, calf fecal, cow nasal, cow fecal, and cow vaginal samples, respectively.

**Fig 4:**
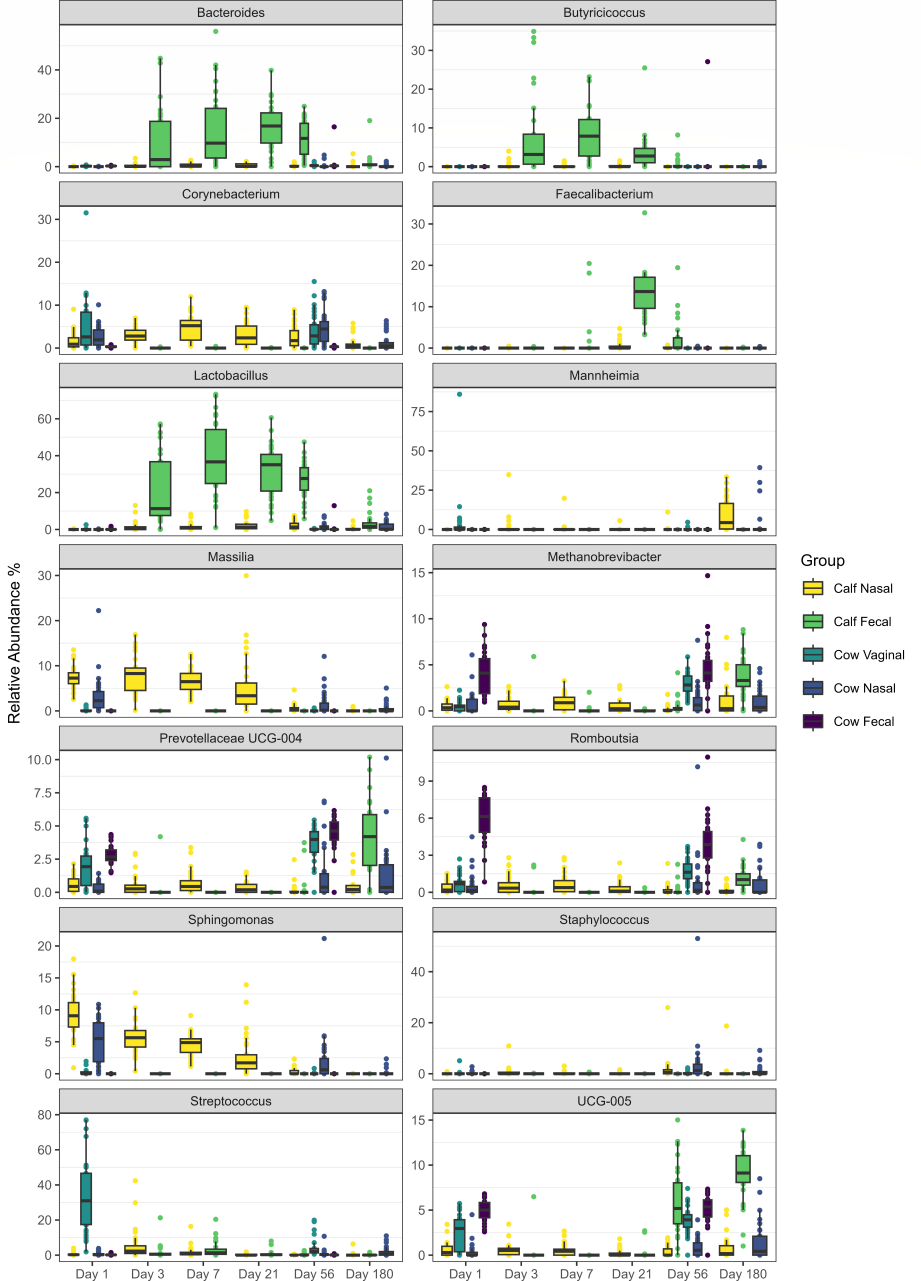
Relative abundance of the 14 most abundant genera in cow and calf samples over time. Calf (*N* = 30) deep nasal swabs and feces, as well as cow (*N* = 30) nasopharyngeal, fecal, and vaginal samples were analyzed over 180 days. Day 1 was the day of birth. Error bars indicate ±standard error of the mean. The box in the plots indicates the interquartile range (IQR) (middle 50% of the data), the middle line represents the median value, and the whiskers represent 1.5 times the IQR.

When evaluated across sample type, the 14 most-abundant genera included *Lactobacillus* (7.6%), *Bacteroides* (3.5%), *Streptococcus* (3.3%), *Oscillospiraceae* UCG-005 (UCG-005; 2.4%), *Massilia* (2.0%), *Corynebacterium* (1.8%), *Sphingomonas* (1.7%), *Methanobrevibacter* (1.5%), and *Prevotellaceae* UCG-004 (1.5%) (Fig. 4). In some instances, these genera were shared across sites to varying extents (e.g., *Oscillospiraceae* UCG-005), whereas others were mostly limited to one or two sample groups (e.g., *Faecalibacterium* in calf nasal and fecal samples).

**Fig 5:**
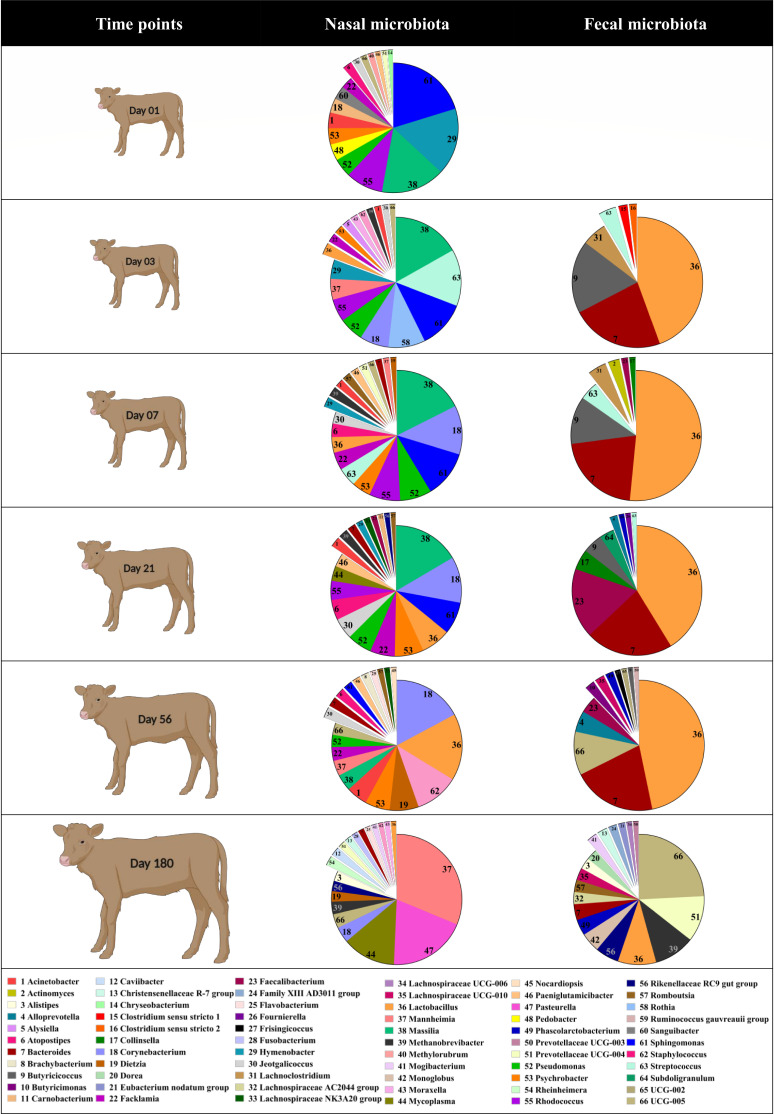
Changes in the respiratory and fecal microbiota of beef calves from birth to weaning. Mean relative abundance of genera present at ≥1% abundance in calf nasal and fecal samples. Deep nasal swabs were collected from calves (*N* = 30) on days 1, 3, 7, 21, 56, and 180, while fecal samples were collected on days 3, 7, 21, 56, and 180. Day 1 was the day of birth. The calf image is created with BioRender.com

When comparing genera between calf and cow nasal samples across time points, 268 genera were shared, while 17 and 316 were only detected in calf and cow nasal samples, respectively (data not shown). In considering only the 15 most relatively abundant genera in calf (Fig. S1) and cow (Fig. S2) nasal samples, 11/15 genera were shared. Although significant changes in the calf nasal microbiota occurred over time (Fig. 5), for some genera, similar changes were apparent in both calf and cow nasal samples. For example, decreases in the relative abundances of *Hymenobacter*, *Massilia*, *Rhodococcus*, and *Sphingomonas* were apparent from days 1 to 180 for both calves and cows (Fig. S1 and S2). While the BRD-associated genus *Mannheimia* was one of the top 15 most abundant genera for both calf and cow nasal samples, the BRD-associated genera *Mycoplasma* and *Pasteurella* were only part of the top 15 for calf nasal genera. Notably, *Histophilus* was not detected as a dominant genus in any respiratory sample. Although five ASVs were classified under the *Histophilus* genus during the taxonomic classification, these ASVs were removed during the ASV filtering step, in which ASVs present in less than 1% of samples were removed from the analysis. Upon manual review, it was determined the *Histophilus* ASVs were present in only a single sample per ASV, leading to their removal.

On the day of birth, it was apparent that the calf nasal microbiota mostly resembled that of the cow nasal microbiota, with the majority of abundant genera sharing similar proportions in respiratory samples from both groups of animals (Table S2). In contrast, there were few similarities to the vaginal tract microbiota, and only three of the top 15 vaginal genera (*Corynebacterium*, *Mannheimia*, and *Streptococcus*; Fig. S3) were common between calf nasal and cow vaginal samples. Despite *Streptococcus* accounting for 53% of the microbial population in vaginal samples, the abundance of *Streptococcus* in calf nasal samples (0.94%) was more similar to that of cow nasal samples (1.6%). A notable exception in similarities between calf and cow nasal samples, and vaginal samples, on the day of birth was *Mannheimia*. This genus was not detected in cow nasal samples on day 1, though it was prevalent in cow respiratory samples on days 56 and 180. In contrast, *Mannheimia* accounted for 0.007% and 4.7% of the microbial populations in calf nasal and cow vaginal samples, respectively, on day 1. Although the *Mannheimia* ASVs could not be differentiated at the species level, interestingly, *Mannheimia* ASV_46 was detected in only calf nasal samples, up to day 56, and cow vaginal samples on day 1 (Fig. S4). On day 180, there was a shift in *Mannheimia* ASV, and ASV_52 became dominant in calf nasal samples, while ASV_114 became dominant in cow nasal samples. While the abundance of *Mannheimia* did not differ between calf and cow sample types, a total of 18 genera were significantly different when comparing cow and calf respiratory samples (*P* < 0.05). Of these, it was notable that *Mycoplasma* and *Pasteurella* were strongly reduced in abundance in calf nasal samples on day of birth, which reversed on day 180, when these two genera became present in greater amounts in calf versus cow nasal samples (Fig. S5).

Similar to respiratory samples, the composition of microbiota in calf feces changed over time (Fig. 5). When comparing genera across time, between calf and cow fecal samples, 100 genera were shared, while 80 were specific to cow fecal samples and 28 were specific to calf feces (data not shown). Unlike respiratory samples, few of the top 15 most abundant genera were common between calf (Fig. S6) and cow (Fig. S7) samples. Only *Methanobrevibater*, *Phacolarctobacterium*, *Prevotellaceae* UCG-004, *Rikenellaceae* RC9 gut group, and *Oscillospiraceae* UCG-005 commonly occurred within the most abundant genera. A total of 36 genera were different in abundance between calf and cow microbiota, with the majority of these being reduced in calf feces (*P* < 0.05; Fig. S8). Cow fecal (Fig. S7) and vaginal (Fig. S3) samples shared 11 of the 15 most relatively abundant genera.

### Changes in calf microbiota longitudinally

**Fig 6:**
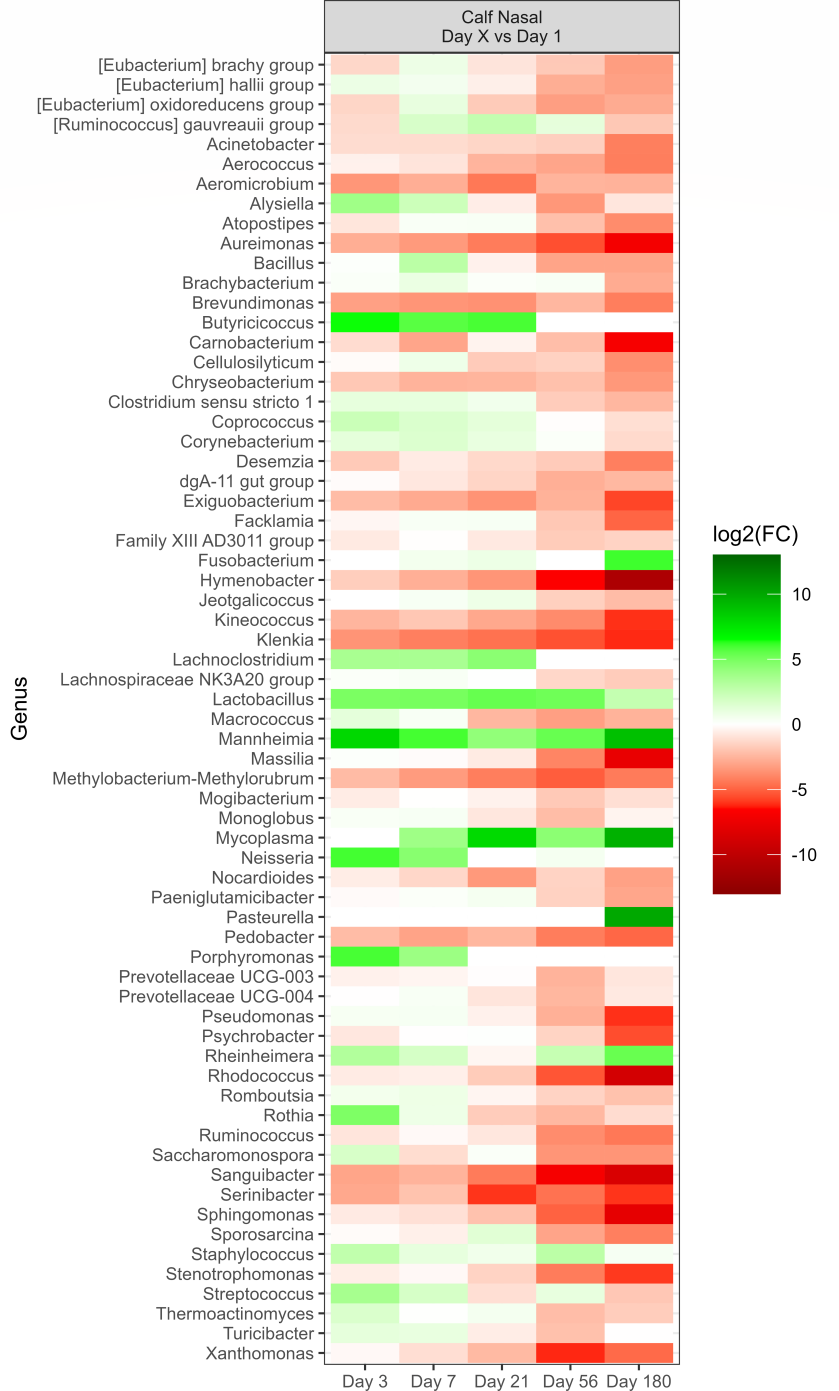
Genera of calf (*N* = 30) deep nasal samples that showed a significant change (*P* < 0.05) with respect to each sampling time against the baseline time (day 1, day after birth). The colors displayed represent the average log2(FC) of amplicon sequence variants (ASVs) with a significant change (*P* < 0.05) within the respective genus at the indicated time.

Across all sampling times of the calf nasal microbiota, there were 67 genera that exhibited a change [*P* < 0.05, log2(FC) > 2 or log2(FC) < −2] in their relative abundance compared to the initial sampling on day 1, as shown in Fig. 6. All of the 15 most relatively abundant genera (Fig. S1) were part of this list that changed over time. Compared to baseline (day 1), *Mannheimia* and *Mycoplasma* were strongly increased at most of the sampling time points. In contrast, *Aureimonas*, *Hymenobacter*, *Klenkia*, *Rhodococcus*, *Sphingomonas*, and *Sanguibacter* consistently decreased over time. Interestingly, *Fusobacterium* and *Pasteurella* became elevated at day 180 (*P* < 0.05), although there were no notable changes at earlier time points. Genera within the order *Lactobacillales* (lactic-acid-producing bacteria) varied in the extent of change over time. *Aerococcus* continuously became less abundant, while *Lactobacillus* increased in abundance compared to day 1, and remained elevated at subsequent time points. *Streptococcus* was variable, but by day 180, was reduced in abundance compared to day 1.

**Fig 7:**
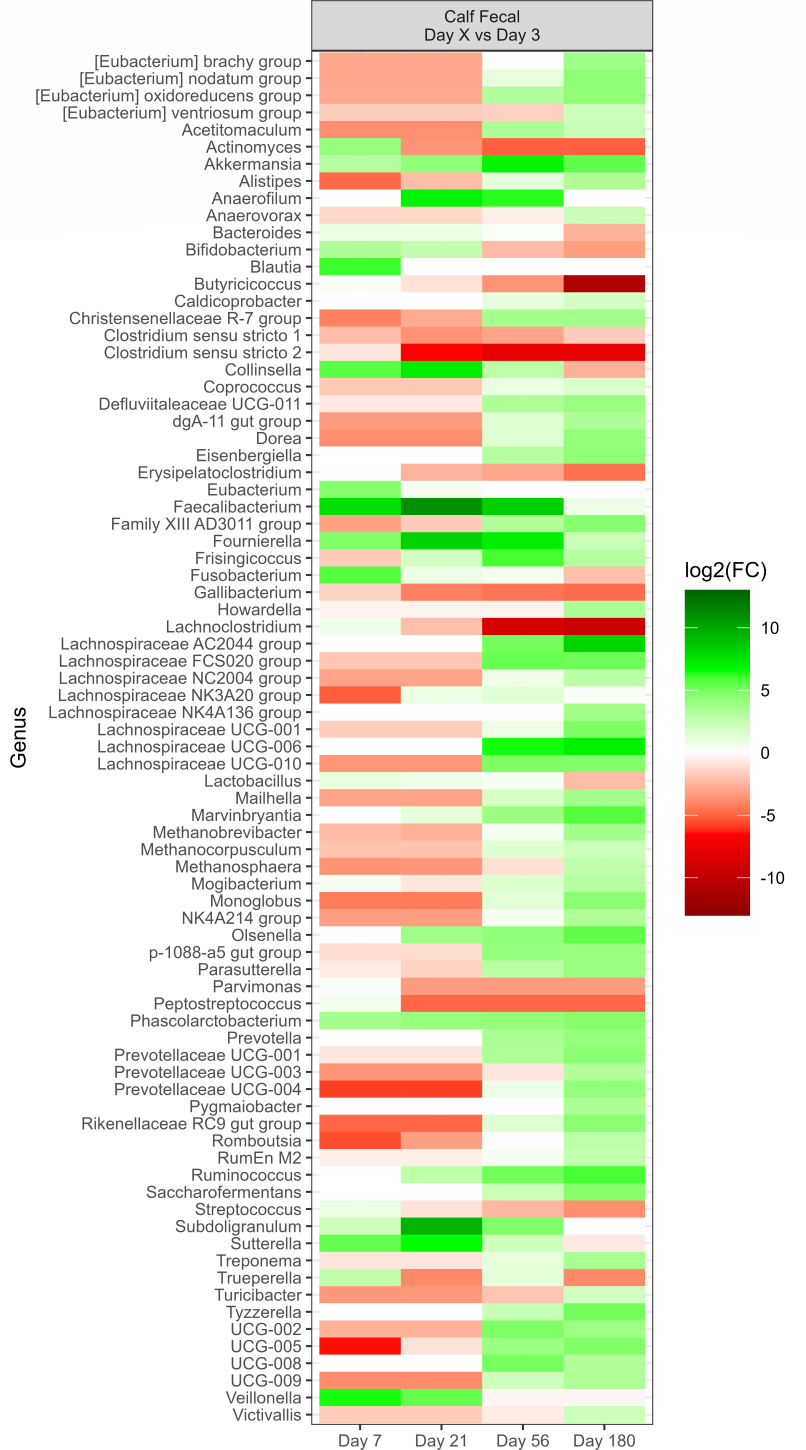
Genera of calf (*N* = 30) fecal samples that showed a significant change (*P* < 0.05) with respect to each sampling time against the baseline time (day 1, first day after birth). The colors displayed represent the average log2(FC) of amplicon sequence variants (ASVs) with a significant change (*P* < 0.05) within the respective genus at the indicated time.

There was a change [*P* < 0.05, log2(FC) > 2 or log2(FC) < −2] in the relative abundance of 80 genera in the calf fecal microbiota, compared to the initial sample taken on day 3, after birth, across all the sampling times (Fig. 7). Each of the 15 most relatively abundant genera (Fig. S7) was included in genera that changed. *Ruminococcus*, *Olsenella*, *Lachnospiraceae* UCG-006, and *Lachnospiraceae* AC2044 genera strongly increased at most of the time points in comparison to the baseline (day 3). Although *Prevotellaceae* UCG-004, *Rikenellaceae* RC9 gut group, *Romboutsia*, UCG-002, and *Oscillospiraceae* UCG-005 showed an initial decrease compared to the baseline, the abundance of these genera gradually increased over time.

### Relationship among calf nasal and fecal microbiota

**Fig 8:**
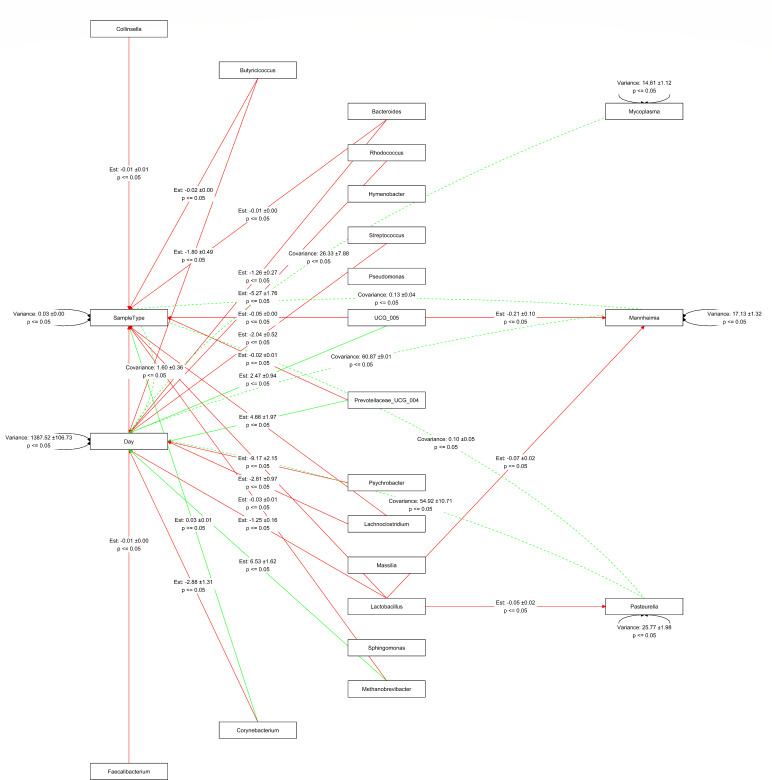
Path analysis of the most abundant genera in calf nasal and fecal samples. Only significant (*P* < 0.05) relationships between variables are represented with paths. Solid lines represent regression estimates between the covariances of variables, with dotted lines representing the covariance of dependent variables. Green represents a positive relationship, while red represents a negative relationship. Variances of the dependent variables are present as black lines referring to the dependent variable.

Modeling of the relationships between the 10 most abundant microbiota in calf nasal and calf fecal microbiomes (20 genera total) was conducted (Fig. 8). A variance-covariance matrix was constructed from the sample group, day sampled, and relative abundances of the 20 genera, including *Mannheimia*, *Pasteurella*, and *Mycoplasma*. Regressions on this matrix produced variable relationships, with sample group, day sampled, and relative abundances of *Mannheimia*, *Pasteurella*, and *Mycoplasma* selected as dependent variables and the 17 other highly abundant genera were selected as independent variables. Sample day and type affected most of the genera in the model, including *Mannheimia* and *Pasteurella* (*P* < 0.05). Of the respiratory genera in the model, *Lactobacillus* was found to have a negative effect on *Mannheimia* (*P* < 0.05; −0.41 ± 0.22; data not shown). Similarly, fecal *Oscillospiraceae* UCG-005 demonstrated a significant negative effect on *Mannheimia* (*P* < 0.05; −0.21 ± 0.10). While examining the combination of fecal and respiratory samples, the model found that *Lactobacillus* has a negative effect (*P* < 0.05) on *Mannheimia* (−0.07 ± 0.02) and *Pasteurella* (−0.05 ± 0.02). Analysis of only calf nasal samples found no link between *Mannheimia* and *Oscillospiraceae* UCG-005 (data not shown), while independent analysis of calf fecal samples was not possible due to the lack of *Mannheimia*, *Pasteurella*, and *Mycoplasma* counts in the fecal data. This indicated that fecal *Oscillospiraceae* UCG-005 had a negative effect on the *Pasteurellaceae* genera in the model.

## DISCUSSION

Understanding how the microbiotas of beef calves change from birth to weaning is important, due to beef cattle being most susceptible to BRD shortly after weaning. While studies have shown that the respiratory microbiota of beef cattle changes after feedlot placement (11, 32, 33), none have described the progression from birth. The ADRI herd was selected for this study due to it being closed, pathogen-free, unvaccinated, and having well-documented parity histories. These factors made the ADRI herd an ideal choice for analyzing microbial changes, as their microbiomes have been minimally affected by external factors like vaccination or antimicrobials (12). A limitation of this study was that cows and calves were placed in uncovered pens for the first 56 days, which is typically not undertaken in commercial farm settings. This was done for ease of sampling and to minimize stress due to movement during the period when sampling was most intense. However, despite this, calves were exposed to ambient conditions and the pens were directly adjacent to fields that animals were transferred to after sampling on day 56.

### The fecal microbiota

Microbial diversity of cow feces remained similar from days 1 to 56, and inter-animal variation was low, indicating a stable microbiota. In contrast, calf fecal microbial diversity increased from birth to weaning, highlighting that the microbiota continued to change and increase in complexity up to day 180. Similarly, in dairy calves, fecal diversity increased from 3 to 35 days of age (34). Although we did not observe calf fecal diversity to stabilize throughout the duration of our study, this point likely occurs sometime after weaning. In dairy calves, fecal microbial diversity increased up to 1 year of age before stabilizing (35), while in beef calves, fecal microbial diversity has been shown to increase for 3 months after weaning (36).

The structure of the calf fecal microbiota shifted as the animals aged, becoming increasingly dissimilar from day 3, across time. Recent studies have shown that the gut microbiota is present in ruminants during the fetal period, with bacteria being detected in the rumen, caecum, meconium, and amniotic fluid at 5 months of gestation and also at birth (37, 38). The source of these bacteria may originate from the dam’s oral microbes (39). In our study, source-tracking analyses revealed a stronger contribution of cow nasal microbiota to calf fecal microbiota composition, compared to other maternal sources, on day 3. Whether transfer occurred *in utero* was not evaluated, but it is likely that direct cow-calf contact and licking were the main contributing factors for this microbiota transmission. It was not possible to collect feces on day 1, due to lack of sample upon digital palpation. However, like our study, others have shown that the gut microbiota of calves becomes diverse and undergoes rapid changes in early life (39–42).

Across the sampling time points, the dominant phyla identified in calf feces were Firmicutes, Bacteroidota, Proteobacteria, and Actinobacteriota, which is consistent with previous reports on dairy (43, 44) and beef calves (45). However, at the genus level, differences existed across these studies. During the first week of life, we observed the calf fecal bacteria to be dominated by *Lactobacillus*, *Bacteroides*, *Butyricicoccus*, *Lachnoclostridium*, and *Streptococcus*. Notably, *Lactobacillus* dominated the microbiota to day 56, which corresponds to when milk consumption and digestion would have been greatest. Previously in beef calves, it was shown that *Oscillospiraceae* UCG-005 and *Lactobacillus* were dominant early in life (45), while *Bifidobacterium* (44) or *Prevotella* and *Bacteroides* were shown to be dominant genera in feces of young dairy calves (43). Typically, dairy calves are separated from cows at birth and raised in enclosed pens or hutches, which may be why differences in beef and dairy cattle fecal microbiota were observed.

After Dday 56, *Lactobacillus* lost its dominance to *Oscillospiraceae* UCG-005, *Prevotellaceae* UCG-004, and *Methanobrevibacter* (day 180). Compared to the baseline time point (day 3), *Ruminococcus*, *Olsenella*, *Lachnospiraceae* UCG-006, *Lachnospiraceae* AC2044 group, *Prevotellaceae* UCG-004, *Rikenellaceae* RC9 gut group, *Romboutsia*, UCG-002, and *Oscillospiraceae* UCG-005 increased in abundance on days 56 and 180. Many of these genera are recognized for their involvement in fiber digestion, emphasizing the impact of increasing plant intake while calves were on pasture. Although the rumen is the primary site of fiber digestion, the hindgut can account for up to 27% and 40% of cellulose and hemicellulose fermentation, respectively (46). Because many of the same microorganisms reside in the rumen and hindgut (47), the establishment of a fiber-digesting community in the lower gastrointestinal tract is also critical to calf health and performance.

### The respiratory microbiota

Similar to fecal samples, the cow nasal microbiota remained relatively stable across the time points evaluated. However, there was a greater extent of variation in both alpha and beta diversity parameters in cow nasal microbiota, compared to cow feces. This variation was likely due to continuous exposure of the upper respiratory tract to environmental bacteria, that altered the composition in individual animals (11). Unlike the calf fecal microbiota, the diversity of the calf nasal microbiota gradually decreased as the calf aged. Diversity was similar during sampling for 1 week after birth, before decreasing on days 21 and 56, and remaining lower on day 180. This reflected the establishment of prominent taxa colonizing the calf respiratory tract after day 56. These results are in contrast to a study that evaluated the respiratory microbiota of dairy calves. Lima et al. (34) observed Shannon index to decrease from days 3 to 14 after birth, then increase again by day 35 (34). In that study, calves were removed from cows at parturition and placed in indoor pens, emphasizing how differences in environment may influence the respiratory microbiota of calves.

The calf nasal microbiota on the day of birth was highly similar to that of cows, in both composition and abundance of the most dominant genera. This was supported by both DCA clustering, FEAST analysis and ANOSIM comparisons between calf and cow nasal samples. Source-tracking analysis of calf nasal microbiota revealed that cow nasal microbiota was the greatest bacterial contributor and it remained higher compared to other cow niches until day 56. Therefore, contact with bacteria harbored in the cow upper respiratory tract had a substantial impact on the colonization and establishment of the nasal microbiota of calves. These findings disagree with those of Lima et al. (34), who reported that cow vaginal microbiota mainly influenced the initial bacterial colonization of the upper respiratory tract of dairy calves (34). As mentioned, in the study by Lima et al., calves were separated from dams at parturition and housed in pens, thus limiting maternal licking and contact with the dam’s upper respiratory tract microbiota (34). Thus the bovine respiratory microbiota can be influenced by practices employed in different management systems. In addition, colonization of the bovine respiratory tract in neonates is responsive to bacteria that make contact early in life. In both our study (up to 180 days of age) and that by Lima et al. (up to 35 days of age) (34), it was shown that the influence by either cow respiratory or vaginal microbiota persisted. Thus, early life intervention may offer an ideal opportunity to alter the respiratory microbiota of calves and enhance health.

The cow vaginal microbiota was more similar to that of cow feces, which has previously been attributed to the proximity of the digestive tract, and bacterial transfer from it, to the reproductive tract (48, 49). Thus, the cow vaginal microbiota appeared to have limited impact on the calf respiratory microbiota in our study, though two exceptions were observed. On day 1, the cow nasal and vaginal relative abundances of *Moraxella* were similar, and it was not possible to estimate which of these niches contributed to *Moraxella* colonizing the calf respiratory tract. Second, *Mannheimia* was detected in only cow vaginal and calf nasal samples on day 1. Although *Mannheimia* was not detected in cow nasal samples on day 1, this was likely due to a low relative abundance, and not the genus being absent in cow respiratory samples, as it was detected at later time points. Given the importance of *Mannheimia* in BRD, we further characterized sequences within this genus at the ASV level. While multiple ASVs within *Mannheimia* were detected in respiratory samples, *Mannheimia* ASV_46 dominated calf samples up to day 56, and this same ASV was the most dominant in cow vaginal samples. On day 180, the relative abundance of *Mannheimia* increased substantially in calf and cow nasal samples, but this was attributed mainly to ASVs 52 and 114, respectively, which were not detected in cow vaginal samples.

*M. haemolytica* primarily colonizes bovine and ovine respiratory tracts (50), and in North America, serotypes 1, 2, and 6 are the dominant strains observed in cattle (51). However, *Mannheimia granulomatis* has been isolated from the intestinal tract of cattle (50); thus, it is plausible that this species also resides within the reproductive tract. Although speciation was not possible by ASV assessment, it is tempting to speculate that a different species or strain of *Mannheimia* originating from the cow reproductive tract colonized calves up to at least 8 weeks of age. Individual ASVs for a single genus may represent a strain or species (52), or potentially be used as an indicator of health status (53). In the study by Lima et al. (34), *Mannheimia* was readily abundant in calf respiratory and cow vaginal samples. Interestingly, in that study, *Mannheimia* was more prevalent in vaginal samples from cows whose calves did not develop pneumonia or otitis. The authors proposed that the calves exposed to a greater abundance of *Mannheimia* were naturally immunized against subsequent infection. Our data suggest that it may also be possible that certain strains of *Mannheimia* outcompeted *M. haemolytica* in the respiratory tract of calves in the first 8 weeks of life. In support of this, we have observed that *M. haemolytica* serotype 1 outcompetes serotype 2 *in vitro* (data unpublished). Thus, bacterial competition within the calf respiratory tract contributes to shaping the community structure.

Previous studies have shown the respiratory microbiota of healthy beef calves to vary, but to mainly be comprised of *Mycoplasma*, *Moraxella*, *Pasteurella*, *Mannheimia*, *Acinetobacter*, *Corynebacterium*, and *Histophilus* (11, 33, 54, 55). These studies support our results, which also showed those genera comprising the majority of sequences in nasal samples. *Histophilus* was mainly absent from the microbiota of all cattle. It has been shown that colonization by *H. somni* is more likely to occur later in the feedlot feeding period (7, 51). These data suggest that colonization by *Histophilus* is largely limited in closed herds, though the reason for this is unknown. The genera *Hymenobacter*, *Massilia*, and *Rhodococcus* were highly abundant at birth, then decreased substantially by days 56–180. These bacteria have in common that they are typically found in environmental sources, including plants and soil (56–59). Thus, being widely prevalent in the calves’ environment likely facilitated colonization early on, followed by reduction once a stable microbiota became established.

Compared to day 1, the structure of the calf respiratory microbiota became increasingly dissimilar across time, as indicated by ANOSIM and DCA plot evaluation. However, the overall structure of the microbiota remained highly similar to the cow’s nasal microbiota for most time points. An exception was on day 180, when the ANOSIM *R*-value increased, and clustering with cow nasal samples in DCA plots became weaker. Interestingly, these changes were driven by increases in genera typically associated with BRD, including *Mannheimia*, *Pasteurella*, and *Mycoplasma*. The relative abundances of both *Pasteurella* and *Mycoplasma* were greater in the calf than cow nasal samples on day 180. While stress has typically been associated with pathogen proliferation in the upper respiratory tract (60), the calves in our study did not experience stressful management practices prior to sampling on day 180, and were weaned immediately after the samples were collected. While reasons for this are unknown, it is noteworthy that *Streptococcus* and *Lactobacillus*, which are lactic acid-producing bacteria, also decreased in relative abundance on day 180. Cattle respiratory strains from both these genera have previously been shown to inhibit the growth of *M. haemolytica*, and their corresponding families, *Streptococcaceae* and *Lactobacillaceae,* were negatively correlated with *Pasteurellaceae* which includes *Mannheimia* and *Pasteurella* (61). Thus, it is possible that as BRD-associated genera increased, bacteria shown to inhibit their growth previously, decreased in the calf respiratory tract prior to weaning.

### Association between gut and respiratory microbiota

The lung microbiota has been shown to be influenced by bacteria in the gastrointestinal tract (8). This is thought to occur from enteric bacteria producing metabolites that modulate lung immunity and inflammation, or the transfer of immune cells from the gut to the lung (8). Although we did not sample the lungs of calves, we predicted that factors affecting lung innate immunity and microbiota may also have similar impacts on the upper respiratory tract. The nasopharyngeal microbiota has previously been shown to be highly related to the lung microbiota of beef cattle (62). We used path analysis to model the interrelationships of selected bacterial genera from both the calf nasal and fecal microbiotas, in an effort to measure microbial effects on the BRD-associated genera *Mannheima*, *Pasteurella*, and *Mycoplasma*. Path analysis can help predict potential mutualistic and antagonistic interactions among bacterial species (63). The relative abundance of both *Mannheimia* and *Pasteurella* was negatively affected by *Lactobacillus* in the nasal samples. This is in agreement with previous studies showing that *Lactobacillaceae* was negatively associated with *Pasteurellaceae* (16), and *Lactobacillus* strains inhibited *M. haemolytica in vitro* (64) and *in vivo* (17). Thus, *Lactobacillus* is likely an important antagonist of *Pasteurellaceae* in the bovine respiratory tract.

It was observed that fecal *Oscillospiraceae* UCG-005 negatively affected *Mannheimia*. This same genus has been associated with asthma in children, where low fecal abundance was related to increased wheeze frequency later in life (65). *Oscillospiraceae* UCG-005 has also been shown to have a possible role in calf gut health. Fan et al. reported a network analysis where *Oscillospiraceae* UCG-005 was an integral hub among 20 core fecal bacteria, and was negatively associated with bacteria enriched in watery feces of calves (45). *Oscillospiraceae* UCG-005 may therefore have an effect on both gut and respiratory health, potentially through short-chain fatty acid production by this acetate-producing group (66). Short-chain fatty acids produced by bacterial metabolism of fiber can travel to the bone marrow after absorption and influence the growth of immune cells that participate in respiratory immunity (8). Potentially, *Oscillospiraceae* UCG-005 may have negatively affected respiratory *Mannheimia* through this mechanism. While further studies are needed to confirm this association, our model predicted that a member of the gut microbiota may be involved in modulating respiratory bacteria in cattle. It has been shown that certain gut bacteria in the hindgut of cattle were associated with IgG levels in blood plasma, indicating that gut bacteria may have an effect on immunity at other sites (36). Thus, while preliminary in nature, evaluation of a potential bovine gut-lung axis may provide insight into novel methods to mitigate BRD.

### Conclusion

The nasal microbiota of beef calves closely resembles that of cows after birth, in both composition and abundance of the most dominant genera. Microbial source-tracking analysis also indicated that the nasal microbiota of cows influenced the initial composition of both respiratory and fecal microbiota in calves, early in life. Thus, the interaction between dam and calf plays a significant role in the establishment and colonization of the calf’s microbiota, and is influenced by bacteria present in the dam’s upper respiratory tract. The structure of the microbial community in the nasal passages of calves remained similar to that of the cows, until day 180, when it diverged. This was mostly driven by a reduction in *Lactobacillus* and an increase in BRD-associated genera like *Mannheimia* and *Pasteurella*. Diversity of the calf’s fecal microbiota increased with age, becoming more complex. In calf feces, several genera including, *Lactobacillus* and *Bacteroides*, were dominant in the first 8 weeks of life, but were then replaced by fiber-digesting bacteria as calves transitioned to increased plant intake. When the respiratory and fecal microbiotas of calves were analyzed together, *Lactobacillus* and *Oscillospiraceae* UCG-005 had negative effects on respiratory *Mannheimia* or *Pasteurella*, indicating a potential link between gut and respiratory health in cattle. Overall, both respiratory and gut bacteria represent potential targets to enhance the respiratory health of calves prior to feedlot placement.

## Data Availability

The raw sequencing data employed in this article have been submitted to the NCBI Sequence Read Archive (SRA) under BioProject accession ID PRJNA1014493 (http://www.ncbi.nlm.nih.gov/sra).

## SUPPLEMENTAL MATERIAL

Supplemental figures msystems.00238-24-s0001.pdf Figures S1-S8.

Supplemental tables msystems.00238-24-s0002.xlsx Tables S1 and S2.

ASM does not own the copyrights to Supplemental Material that may be linked to, or accessed through, an article. The authors have granted ASM a non-exclusive, world-wide license to publish the Supplemental Material files. Please contact the corresponding author directly for reuse.
