# Supplementary material for: The respiratory and fecal microbiota of beef calves from birth to weaning
Source: mSystems. 2024 Jun 20;9(7):e00238-24. doi: 10.1128/msystems.00238-24 (PMC11264934; doi:10.1128/msystems.00238-24)
Supplement: Supplemental figures — Figures S1-S8. [file msystems.00238-24-s0001.pdf]

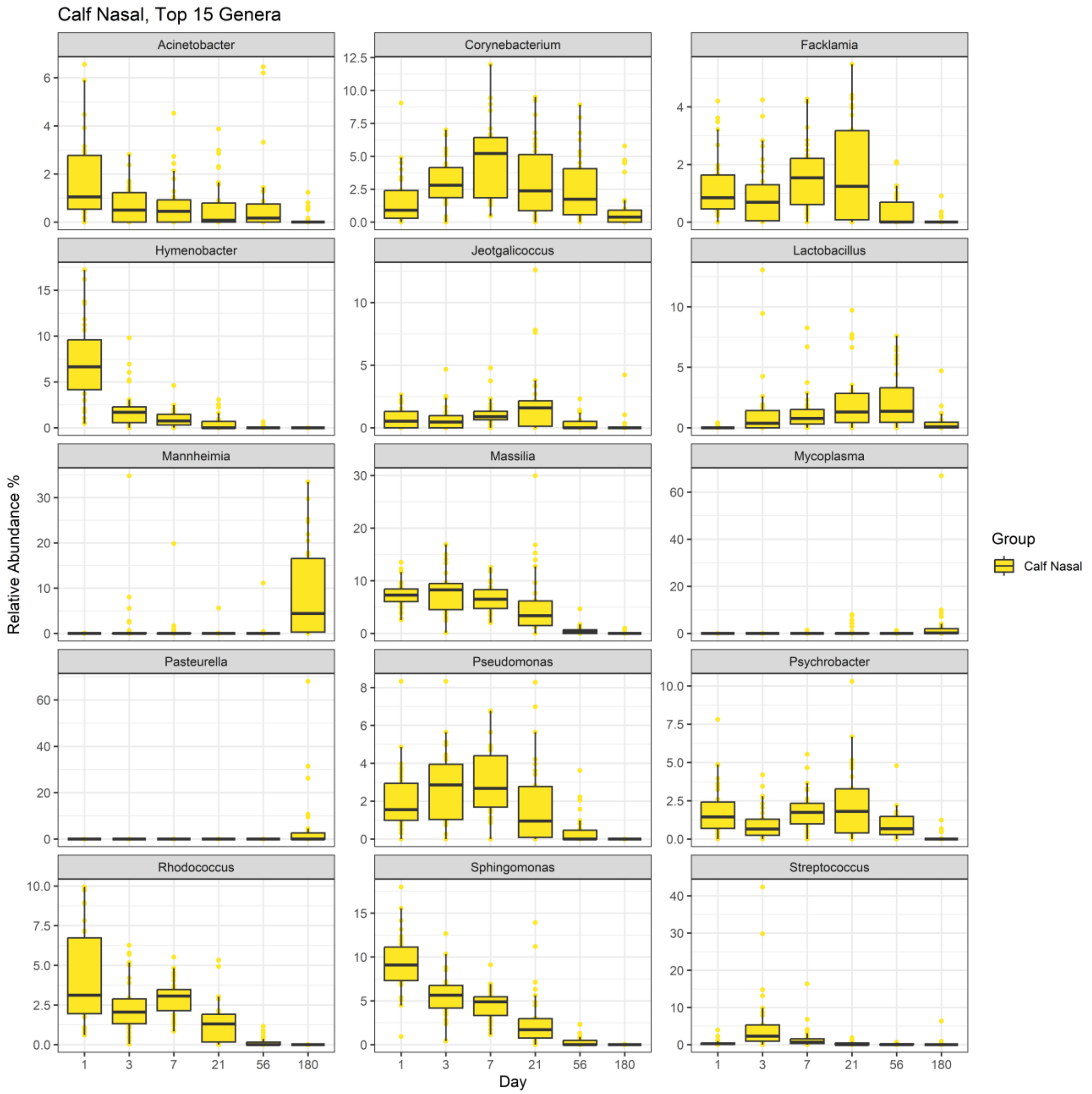

Supplemental Figure 1: Relative abundance of the fifteen most abundant genera in calf (N=30) deep nasal samples over time. Error bars indicate ± standard error of the mean. The box in the plots indicates the interquartile range (IQR) (middle 50% of the data), the middle line represents the median value, and the whiskers represent 1.5 times the IQR.

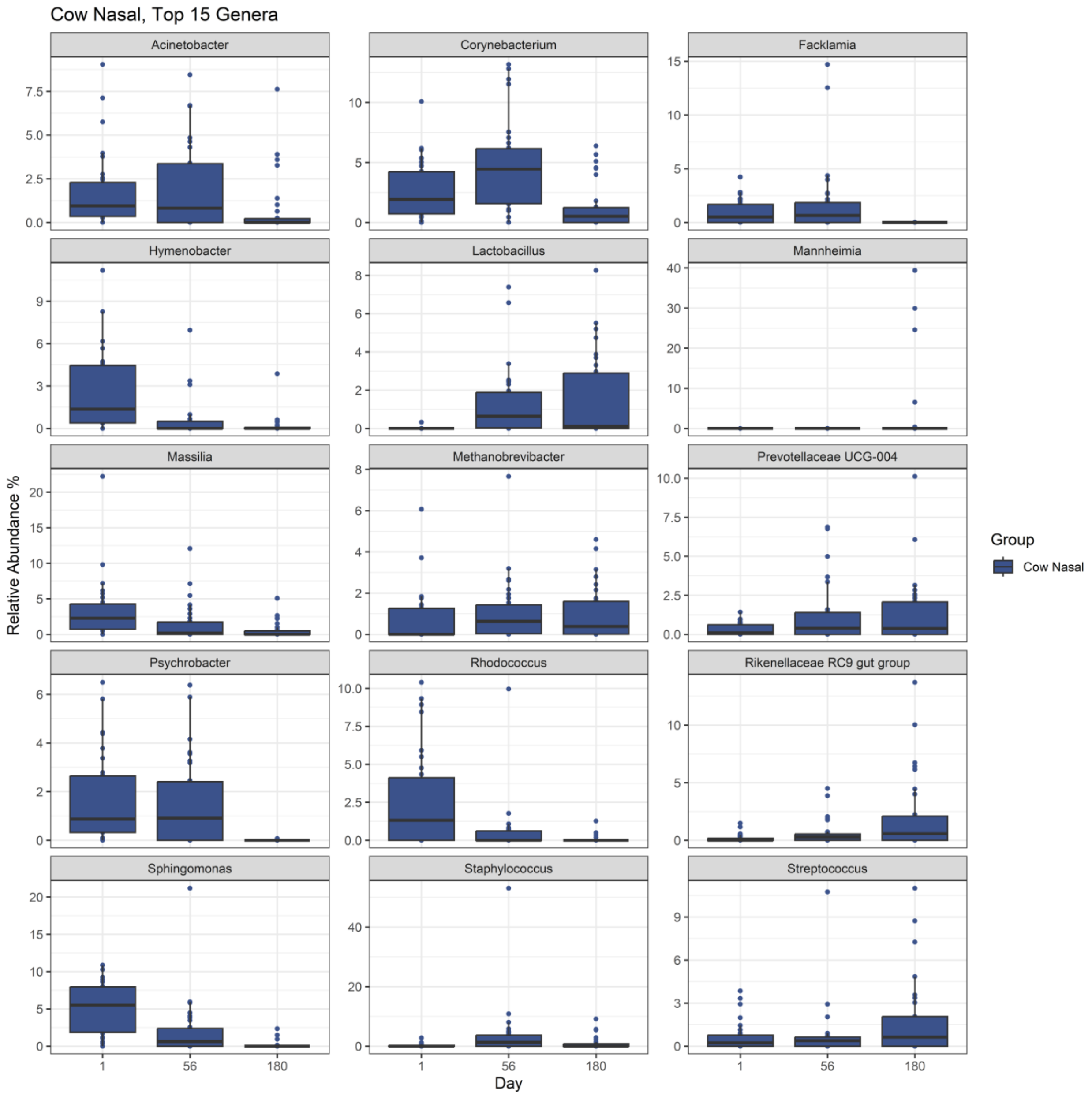

Supplemental Figure 2: Relative abundance of the fifteen most abundant genera in cow (N=30) nasopharyngeal samples over time. Error bars indicate ± standard error of the mean. The box in the plots indicates the interquartile range (IQR) (middle 50% of the data), the middle line represents the median value, and the whiskers represent 1.5 times the IQR.

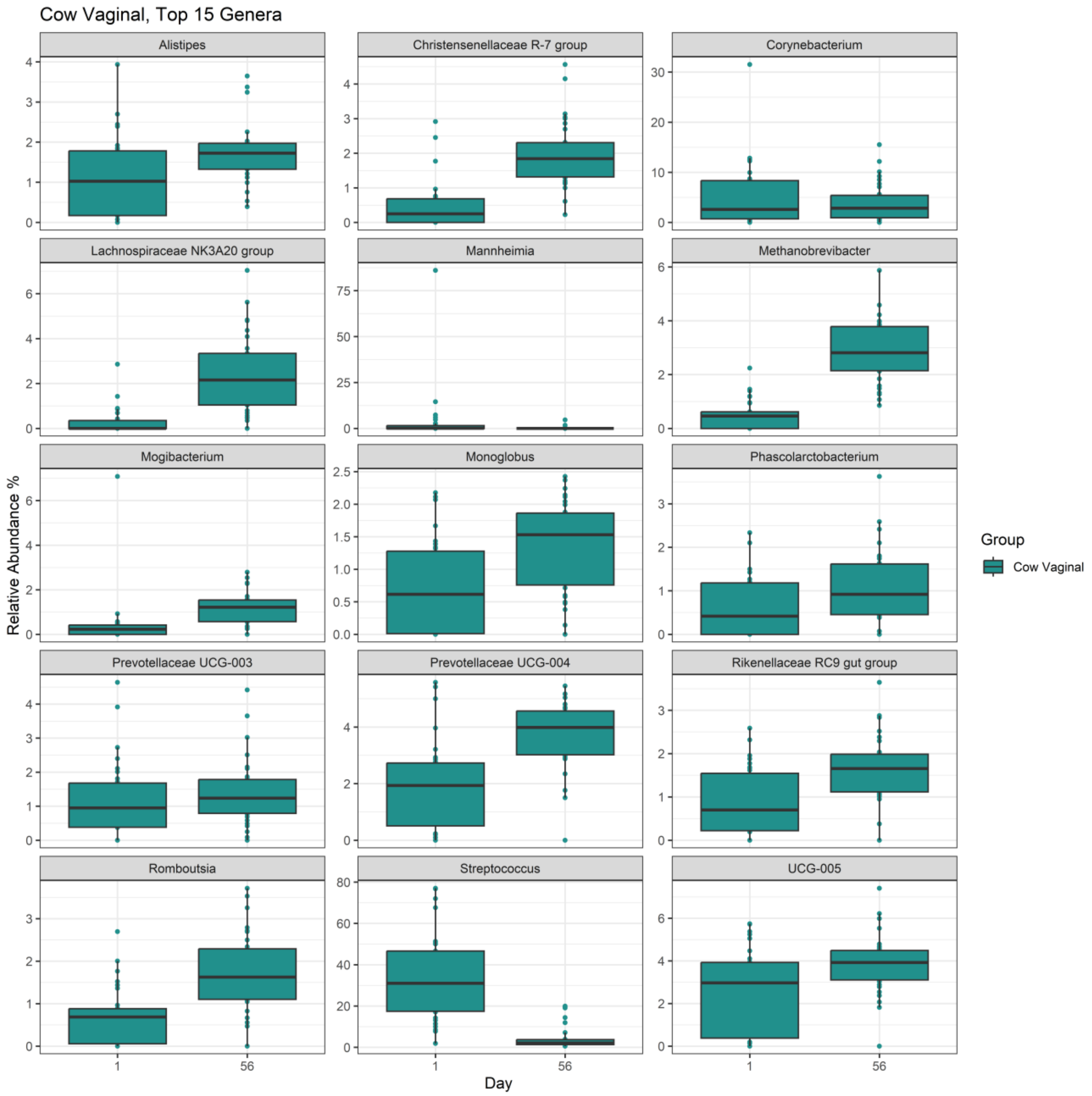

Supplemental Figure 3: Relative abundance of the fifteen most abundant genera in cow (N=30) vaginal samples over time. Error bars indicate ± standard error of the mean. The box in the plots indicates the interquartile range (IQR) (middle 50% of the data), the middle line represents the median value, and the whiskers represent 1.5 times the IQR.

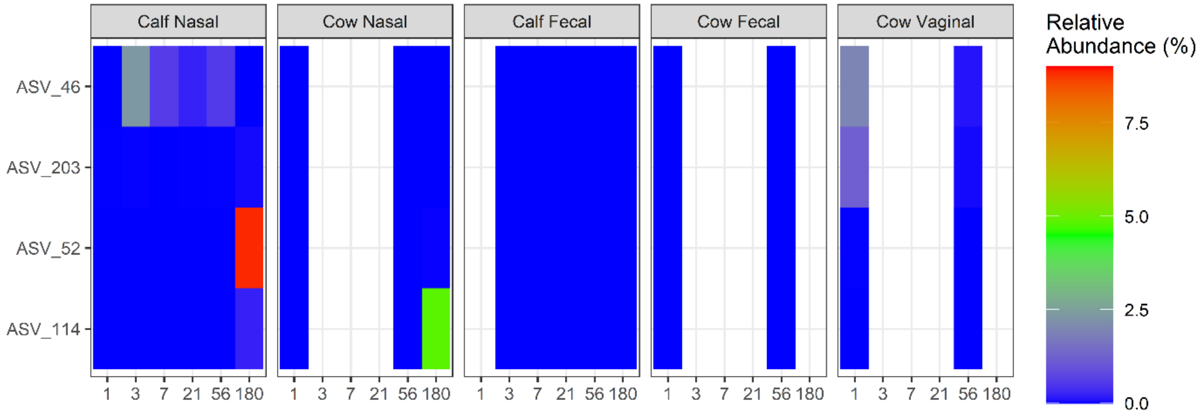

Supplemental Figure 4: Relative abundance of the most dominant *Mannheimia* ASVs in cow (N=30) and calf (N=30) samples over time.

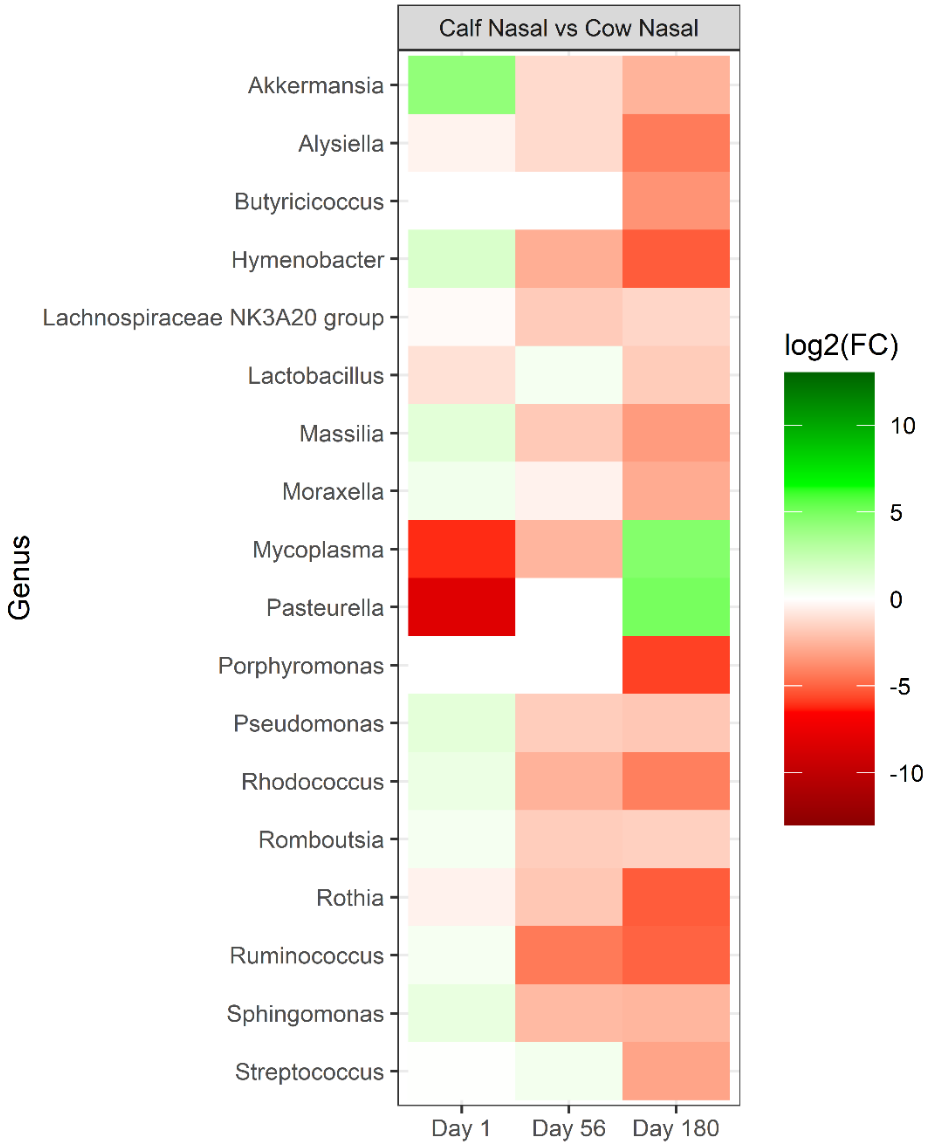

Supplemental Figure 5: Genera of calf deep nasal samples that showed a significant change ($P < 0.05$) against cow nasopharyngeal samples. The colours displayed represent the average log2(FC) of amplicon sequence variants (ASVs) with a significant change ($P < 0.05$) within the respective genus at the indicated time.

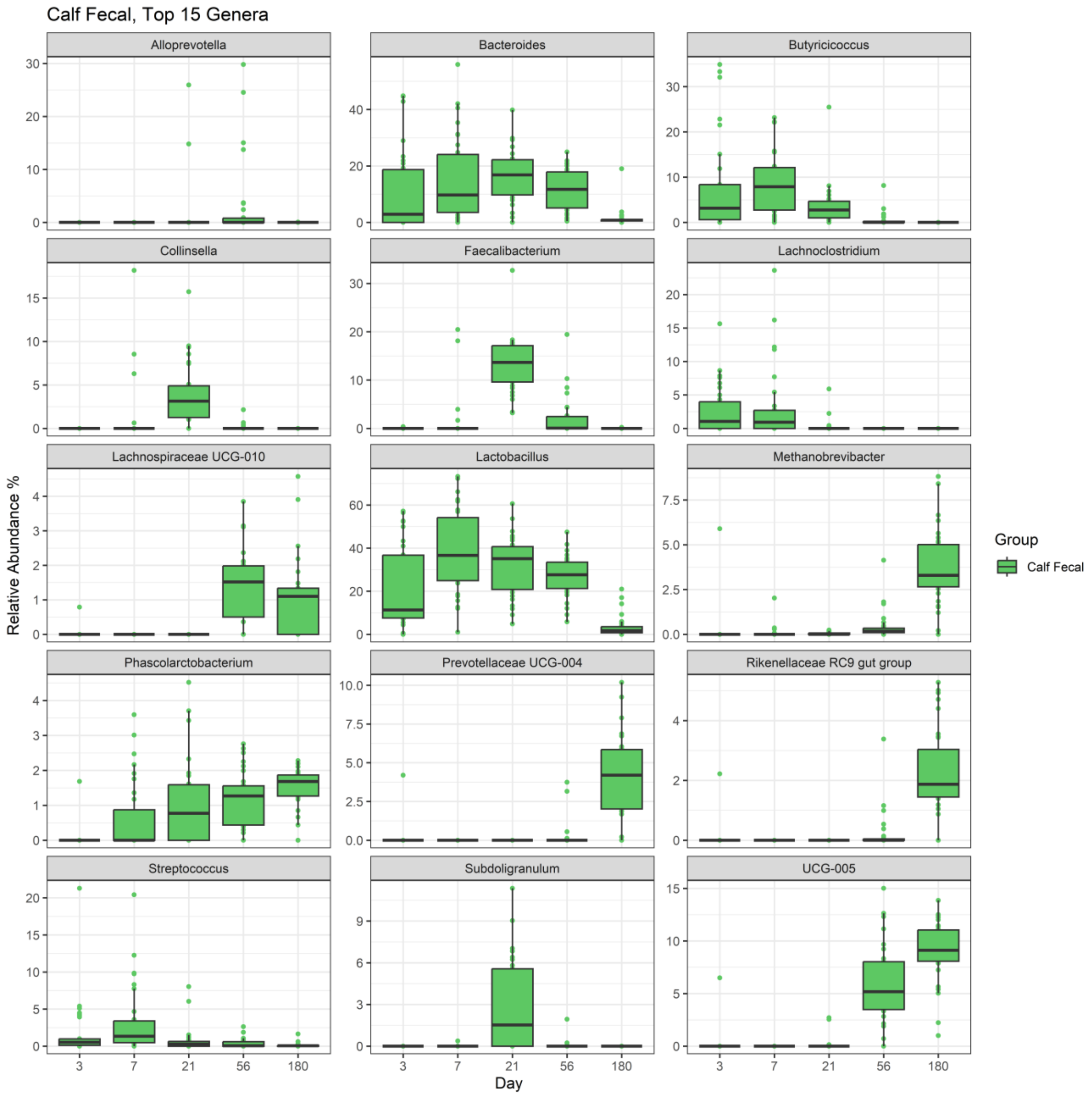

Supplemental Figure 6: Relative abundance of the fifteen most abundant genera in calf (N=30) fecal samples over time. Error bars indicate ± standard error of the mean. The box in the plots indicates the interquartile range (IQR) (middle 50% of the data), the middle line represents the median value, and the whiskers represent 1.5 times the IQR.

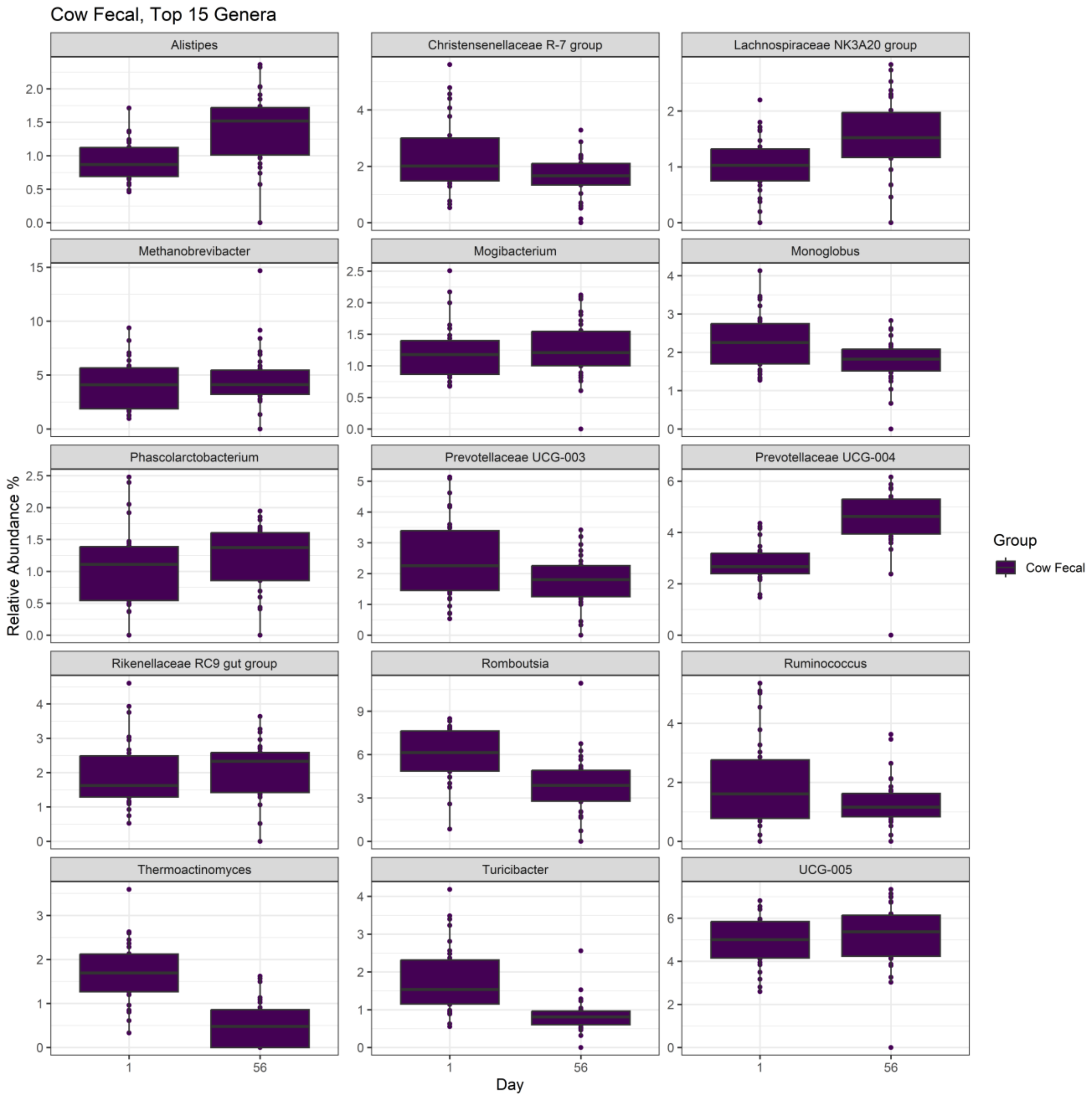

Supplemental Figure 7: Relative abundance of the fifteen most abundant genera in cow (N=30) fecal samples over time. Error bars indicate ± standard error of the mean. The box in the plots indicates the interquartile range (IQR) (middle 50% of the data), the middle line represents the median value, and the whiskers represent 1.5 times the IQR.

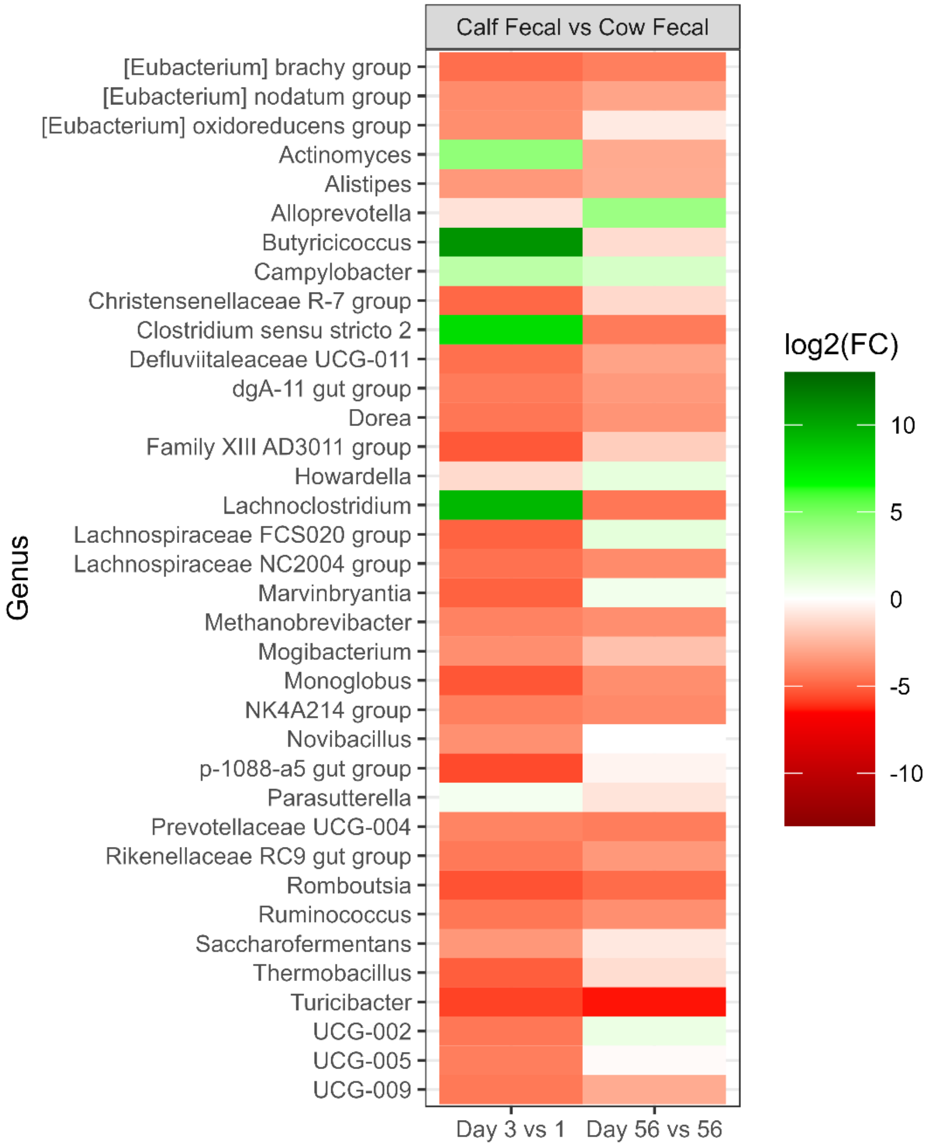

Supplemental Figure 8: Genera of calf (N=30) fecal samples that showed a significant change (P < 0.05) against cow (N=30) fecal samples. The colours displayed represent the average log2(FC) of amplicon sequence variants (ASVs) with a significant change (P < 0.05) within the respective genus at the indicated time.
